# CCL1 is a major regulatory T cell attracting factor in human breast cancer

**DOI:** 10.1186/s12885-018-5117-8

**Published:** 2018-12-20

**Authors:** Benjamin Kuehnemuth, Ignazio Piseddu, Gabriela M. Wiedemann, Michael Lauseker, Christina Kuhn, Simone Hofmann, Elisa Schmoeckel, Stefan Endres, Doris Mayr, Udo Jeschke, David Anz

**Affiliations:** 1 Center of Integrated Protein Science Munich (CIPS-M), Division of Clinical Pharmacology, Klinikum der Universität München, Munich, Germany; 2 Medizinische Klinik und Poliklinik II, Klinikum der Universität München, Munich, Germany; 3 Pathologisches Institut, Medizinische Fakultät der Ludwig-Maximilians Universität München, Munich, Germany; 4 Department of Medicine II, Klinikum Rechts der Isar, Technische Universität München, Munich, Germany; 5 Klinik und Poliklinik für Frauenheilkunde und Geburtshilfe, Klinikum der Universität München, Munich, Germany; 6 Institut für medizinische Informationsverarbeitung, Biometrie und Epidemiologie, Klinikum der Universität München, Munich, Germany

**Keywords:** Regulatory T cells, Breast cancer, Chemokine, CCL1

## Abstract

**Background:**

Regulatory T cells (Treg) suppress cytotoxic T cell anti-tumoral immune responses and thereby promote tumor progression. Prevention of intratumoral Treg accumulation by inhibition of their migration to the tumor microenvironment is a promising therapeutic strategy. The aim of this study was to identify the role of the two major Treg-attracting chemokines CCL1 and CCL22 in human breast cancer.

**Methods:**

One hundred ninety-nine tissue samples of patients with invasive breast cancer were stained for CCL1 and CCL22 by immunohistochemistry. Chemokine expression and tumor infiltration by regulatory T cells, determined by expression of the transcription factor FoxP3, were quantified and their correlation to clinical features was statistically analyzed.

**Results:**

Both CCL1 and CCL22 were expressed in most breast cancer tissues. CCL1 was significantly over-expressed in invasive breast cancer as compared to normal breast tissue. CCL1, but surprisingly not CCL22, showed a significant correlation with the number of tumor-infiltrating FoxP3+ Treg (*p*< 0.001). High numbers of intratumoral CCL1 expressing cells were related to high grade tumors (G4) and a positive estrogen receptor (ER) status whereas high CCL22 expression was generally seen in lower grade tumors. The median survival of 88 patients with high intratumoral CCL1 expression was 37 months compared to 50 months for the 87 patients with low CCL1 levels, this trend was however not statistically significant.

**Conclusions:**

We found a high expression of CCL1 in human breast cancer. CCL1 significantly correlated with the infiltration of immunosuppressive FoxP3+ Treg, that are known to negatively affect survival. Thus, CCL1 may serve as prognostic marker and novel therapeutic target in breast cancer.

## Background

Breast cancer is the most frequent cancer in women worldwide, currently affecting 12% of all women at one time in their life [1]. It is a heterogeneous disease including a wide range of biological behaviors and prognostic characteristics [2]. During the last decades, early diagnosis and novel therapies helped to improve survival rate in breast cancer [2]. However, current therapeutic approaches are still limited and breast cancer still accounts for 14% of cancer-related mortality [3]. In the recent years, with the emergence of checkpoint inhibitors and the possibilities of engineered T cells, cancer immunotherapy has experienced a breakthrough in some tumor entities [4]. Also in breast cancer, checkpoint inhibitors are currently evaluated in several clinical trials and might be effective in a subgroup of patients [5–8]. However, a close understanding of the tumor microenvironment and its mechanistic is required to successfully develop immunotherapeutic strategies in breast cancer.

Regulatory T cells (Treg) are a subtype of immunosuppressive CD4+ T cells that inhibit the cytotoxic function of CD8+ T lymphocytes [9]. The physiological role of Treg is to protect the body from autoimmunity by suppressing self-reactive cells, including CD8+ cytotoxic T cells, B cells and natural killer cells (NK cells) [10, 11]. However, Treg also play an important role in cancer-associated immunosuppression [12]. The presence of Treg in tumor, serum or lymph nodes of cancer patients is related to poor survival in a variety of malignant diseases [13, 14]. In breast cancer, a strong infiltration with CD8+ cytotoxic T lymphocytes has been reported to be associated with a favorable response to neoadjuvant chemotherapy and good clinical outcome in breast cancer [15–17]. By contrast, a high number of Treg has been associated to poor prognosis in different types of breast cancer [18–20]. In order to prevent Treg recruitment to tumor tissues, it is important to identify the mechanisms of Treg attraction. One of the most extensively described mechanisms of Treg attraction to tumor sites is intratumoral expression of the chemokine CCL22 [21].

CCL22 was found in several cancer types, often associated with high infiltration of Treg and low survival [22–24]. Likewise, high expression of CCL22 in breast cancer is related to a higher Treg infiltration and reduced prognosis [25]. Another more recently described chemokine that promotes Treg de novo conversion and also Treg attraction to tumors is CCL1 [26, 27]. It was shown that Sox2-mediated CCL1 expression in murine breast cancer models was related to a higher infiltration by Treg and CCL1 overexpression led to an increase of Treg accumulation [27]. To our knowledge, CCL1 expression in human breast cancer tissues and its relation to Treg infiltration have not been described to date.

In order to determine the role of CCL1 and CCL22 on Treg attraction to breast cancer, we analyzed 199 breast cancer tissue samples that were previously stained [28] for expression of CCL1, CCL22 and FoxP3. Chemokine expression and Treg infiltration were statistically examined for their effects on patient survival. We found a significantly increased expression of CCL1 in breast cancer tissues, which was related to a higher infiltration of Treg. By contrast, expression of CCL22 was not upregulated in tumor tissues compared to healthy breast tissue and showed no impact on Treg infiltration. Our data highlight the role of CCL1 on Treg migration into breast cancer tissue, a finding that might lead to novel therapeutic strategies in breast cancer immunotherapy.

## Methods

### Tissue samples and patient characteristics

All tissue samples derived from female patients diagnosed with mammary carcinoma at the Klinikum der Universität München between 1986 and 2007 (*n* = 199). All patients underwent surgical treatment of either mastectomy or wide local excision with radiotherapy at the local gynecology unit within 7 months after diagnosis. Histological and molecular characteristics of tumors were determined by the local Institute of Pathology according to the current WHO classification. One hundred eighty of the tumors were classified as ductal, 14 as lobular and 5 as unclassifiable. Non-malignant control tissues were obtained from women that underwent breast reduction surgery (*n* = 7).

### Tissue microarray (TMA) and immunohistochemistry

A total of 7 TMA blocks containing 199 consecutive cases were constructed by inserting cylindric tissue cores measuring 2 mm in diameter into a paraffin block. For each tumor and non-malignant tissue 2 cores were embedded. Sections of each TMA block were mounted on silane-coated slides and subsequently further processed for immunohistochemistry as described before [29]. In short, antigen retrieval was performed by 5 min cooking in citric buffer (pH = 6.0). For blocking ZytoChem Plus (HRP) Polymer Kit (Zytomed, Berlin, Germany) was used according to manufacturer’s instructions. Primary antibodies against CCL22 (Peprotech, Hamburg, Germany), FoxP3 (Abcam, Cambridge, USA) and CCL1 (Atlas antibodies, Stockholm, Sweden) were incubated for 16 h at 4 °C. Subsequent to 30 min of incubation with a horseradish peroxidase-polymer (Zytomed, Berlin, Germany) staining was carried out using 3,3-diaminobenzidine-substrate solution (DAB) (Dako, Glostrup, Denmark).

### Statistical analysis

Stained slides were scanned with a high resolution scanner MIRAX MIDI (Zeiss, Jena, Germany). CCL1- and CCL22-positive cells (cytoplasmic staining) as well as FoxP3-positive cells (nuclear staining) were counted independently by two observers (BK and IP).

To calculate the number of stained cells per mm^2^ the area of each core was determined using ImageJ software (V1.50i, NIH, USA). Of the 199 tumors on the array, 180 presented an assessable FoxP3 staining, 175 an assessable CCL1 staining and 174 an assessable CCL22 staining.

The numbers of stained cells per mm^2^ were compared between covariates using the Mann-Whitney-Wilcoxon test. For correlations, the Spearman correlation coefficient was used. Survival probabilities were estimated using the Kaplan-Meier method and compared using the log-rank test. Hazard ratios were derived from the Cox proportional hazards model. *P* values below 0.05 were considered significant. Due to the exploratory character of this work, all *p* values have to be interpreted descriptively.

### Ethics

The restrospectively registered study was approved by the ethics committee of the Ludwig-Maximilians-Universität München.

## Results

### High expression of CCL1 in invasive breast cancer

**Fig. 1:**
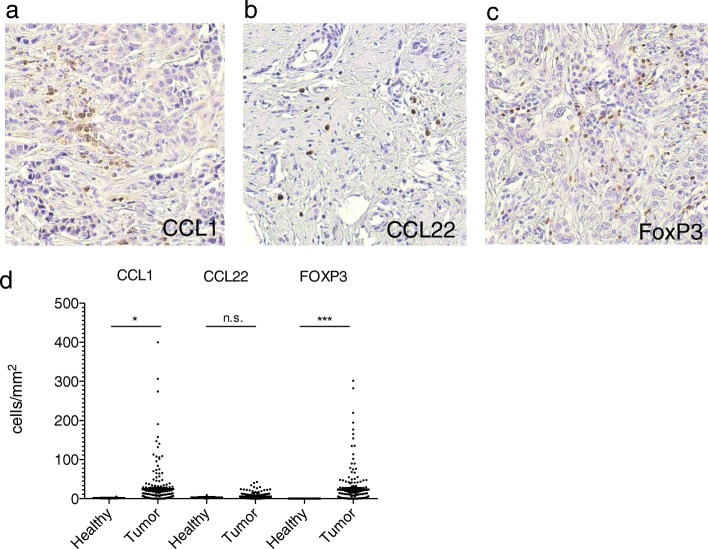
CCL1 and FoxP3 are intensively expressed in human invasive breast cancer. Tissue microarrays (TMAs) of mammary carcinoma and non-malignant control samples have been immunohistochemically stained for the indicated proteins. Representative sections for (**a**) CCL1, (**b**) CCL22 and (**c**) FoxP3 are shown. (**d**) TMAs of breast cancer and healthy breast tissue were analyzed for the number of positive cells/mm^2^. For statistical analysis the Mann-Whitney-U-Test was used

It is well established that high infiltration by FoxP3+ Treg has an adverse effect on prognosis in breast cancer [30]. We have recently described expression of the classic Treg-attracting chemokine CCL22 in breast cancers [31]. CCL1 has been described to be expressed by breast cancer stem cells and has been found to impact Treg migration in murine breast cancer models [27, 32]. We therefore aimed at analyzing the roles of the chemokines CCL1 and CCL22 in human invasive breast cancer. We stained tissue microarrays (TMA) of 199 breast cancer patients for expression of CCL1, CCL22 and FoxP3. We found CCL1, CCL22, and FoxP3 expressing cells in most breast cancer tissues analyzed (Fig. 1a-c). Chemokine-expressing cells were phenotypically not identified as tumor cells but rather tumor-associated immune cells (Fig. 1a-c). Strikingly, we found significantly increased numbers of CCL1 and FoxP3 expressing cells in breast cancer tissues compared to normal breast tissues (Fig. 1d). Although CCL22 has been best described in the context of Treg recruitment to cancer, CCL22 levels were not significantly increased in our breast cancer tissue cohort (Fig. 1d). Taken together, CCL1 expressing immune cells and FoxP3+ Treg are significantly increased in invasive breast cancer tissues compared to healthy breasts.

### Intratumoral expression of CCL1 correlates to a higher infiltration of FoxP3+ Treg

We next sought to evaluate possible associations between chemokine and Treg infiltration. Strikingly, expression levels of CCL1 showed a strong positive correlation with Treg infiltration (Table 1). No significant correlation however was found between CCL22 expression and Treg numbers, indicating that CCL22 is not the major Treg-attracting chemokine in breast cancer. Further, high expression of CCL1 was related to negative ER status as well as higher grade tumors (Table 2).

**Table 1:** Correlation between chemokine expression and density of FoxP3+ cells

Feature	Correlation Coefficient	*p*-value
CCL1 vs. FOXP3	0.42	< 0.001
CCL22 vs. FOXP3	0.07	0.360

Correlation coefficient between chemokine expression and FoxP3 expression was analysed using the Spearman rank correlation coefficient

**Table 2:** Association between chemokine expression and pathological features

Feature		CCL1		CCL22		FOXP3	
		Median	*p*-value	Median	*p*-value	Median	*p*-value
ER-receptor	**–**	12.8	0.029	3.1	0.128	18.0	< 0.001
	**+**	5.6		4.2		4.0	
PR-receptor	**–**	10.9	0.672	3.2	0.121	10.4	0.455
	**+**	9.0		4.0		8.0	
HER2-receptor	**–**	20.5	0.116	3.2	0.082	5.2	< 0.001
	**+**	45.2		4.6		23.1	
Grading	2/3	5.1	0.010	6.2	0.006	4.0	< 0.001
	4	12.8		2.8		17,5	

Associations between chemokine or FoxP3 expression to histopathological features of the tumors, more precisely status of estrogen receptor (ER), progesterone receptor (PR), Her2-receptor and tumor grading, have been analyzed by Mann-Whitney-Wilcoxon test

By contrast, higher expression of CCL22 was found in lower grade tumors. Infiltration of Treg was significantly stronger in high grade tumors and was correlated to negative ER status and positive Her2neu status.

As expected, high grade tumors showed a significantly increased hazard ratio for mortality (1.45 vs. 1.0) (Table 3). Altogether, our data suggest that an increased expression of CCL1 is predominantly found in high grade tumors and is related to infiltration by Treg.

**Table 3:** Multivariate survival analysis for pathological features

Feature		Hazard ratio	95% confidence interval	*p*-value
ER-receptor	**–**	1	0.580 to 1.254	0.419
	**+**	0.853		
PR-receptor	**–**	1	0.704 to 1.538	0.842
	**+**	1.041		
HER2-receptor	**–**	1	0.258 to 1.587	0.336
	**+**	0.640		
Grading	2/3	1	1.049 to 1.993	0.024
	4 vs. 2/3	1.446		

Histopathological features have been analyzed by multivariate analysis with cox proportional hazards regression for mortality

### Effect of intratumoral CCL1 and CCL22 on patient survival

**Fig. 2:**
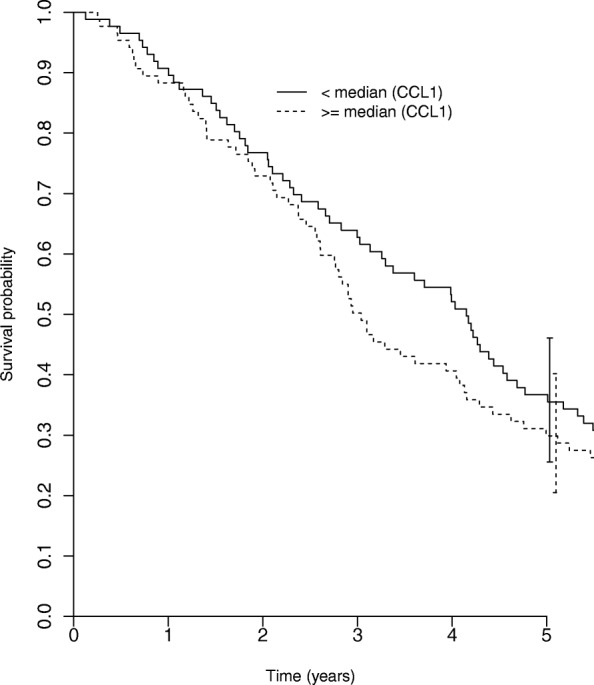
Effect of intratumoral CCL1 on patient survival. Tumor samples have been divided into CCL1 high-expressing (*n* = 88) and CCL1 low-expressing tumors (*n* = 87) based on the median CCL1-positive cell number/mm^2^ (median = 10.14 cells/mm^2^). Survival in both groups was then compared with a log-rank test (*p* = 0.149)

We next analyzed the effect of CCL1 and CCL22 expression on patients’ survival. The statistical follow-up period was 5 years. Expression of CCL22 showed no effect on survival (data not shown). High expression of CCL1 showed some association with poor survival (37 months in CCL1 high vs. 50 months in CCL1 low tumors), however the effect was not statistically significant (HR = 1.1; 95% CI = 0.8–1.5) (Fig. 2). Patients in both groups showed comparable clinical characteristics in terms of UICC stage and age at diagnosis which excluded a potential bias of heterogeneous clinical parameters on the survival rate. UICC stage in the CCL1 high and low group was 1% versus 7% of all patients classified stage I, 44% versus 39% stage II, 19% versus 13% stage III and 35% versus 41% stage IV. The mean patients’ age at time of breast cancer diagnosis was 53,6 years in the CCL1 high compared to 52,8 years in the CCL1 low group respectively.

Thus, in breast cancer, CCL1 rather than CCL22 seems to impact Treg migration and could affect patient survival.

## Discussion

The unfavorable role of Treg in cancers has extensively been demonstrated in the past decades, also in breast cancer. Most publications show a detrimental role on overall survival with high Treg numbers in breast cancer tissues [19, 33]. In order to prevent Treg accumulation at the tumor site, a profound knowledge of the mechanisms of Treg migration is indispensable. In 2004, CCL22 was identified as a Treg attracting chemokine in ovarian cancer [21]. Since then, the role for CCL22 in attraction of CCR4+ Treg to tumors was proven in numerous studies [25, 34, 35]. Other chemokines which have been associated with Treg recruitment to tumors are CCL1, CCL5, CCL17, CCL20 and CCL28, acting on the chemokine receptors CCR4, CCR5, CCR6, CCR8 and CCR10 [22, 36–38]. Amongst these, CCL1 has been described to play a role on Treg de novo conversion and Treg recruitment to murine breast cancer models [32, 39]. CCL1 binds to CCR8, a receptor that is known to be crucial for Treg function and proliferation [39]. In order to investigate the role of CCL1 and CCL22 on Treg infiltration and overall survival in breast cancer patients, we stained tissue microarrays of 199 breast cancer patients for the CCL1, CCL22 and FoxP3. Surprisingly, our data showed upregulation of CCL1 in breast cancer tissues, whereas CCL22 expression was not elevated when compared to normal breast tissue and did not correlate with Treg infiltration.

Chemokine expression and chemokine functions have widely been studied in breast cancer. Chemokines with well-known functions in mammary cancer include CCL2, CCL5, CCL19, CCL20, CCL21 and CCL22 [40]. Their role ranges from angiogenesis and metastasis to attraction of various immune cell subtypes as macrophages, dendritic cells and regulatory T cells [41–45]. By contrast, CCL1 is known to activate Treg and promote FoxP3 expression, de novo conversion and CCR8 expression on Treg [32, 39]. Its role in shaping the tumor microenvironment was recently demonstrated by the fact that CCL1 blockade in murine breast cancer models led to reduced Treg numbers [32]. Moreover, phenotyping of human breast cancer infiltrating Treg revealed high expression of CCR8 as compared to peripheral blood Treg and CCR8 expression on intratumoral Treg had a negative impact on clinical outcome [46]. These data affirm our findings, which identify CCL1 as a major component of the breast cancer immunosuppressive machinery. The fact that high expression of CCL1 and FoxP3 was found in high-grade tumors again suggests their detrimental effect on prognosis, although we could not find a significant effect on overall survival or tumor-free survival in our analysis. We saw a correlation of CCL1 expression to estrogen receptor status, which will be interesting to follow up on in further studies. Considering the heterogeneity of breast cancer, we believe that survival analysis will have to be repeated in a bigger patient cohort, which will facilitate to account for the different breast cancer subtypes. A more extensive analysis might thus unravel the role of CCL1 mediated Treg recruitment on breast cancer patient survival.

## Conclusion

In summary, we identified CCL1 as a major Treg-attracting chemokine in human invasive breast cancer. CCL1 was highly upregulated in breast cancer, positively correlated with Treg infiltration and high grade tumors, whereas none of these was found for CCL22. We conclude that CCL1 might offer new interesting starting points for immunotherapy in breast cancer.

## Abbreviations

CCL: C-C motif chemokine ligand
CCR: C-C chemokine receptor
CI: Confidence interval
ER: Estrogen receptor
FoxP3: Forkhead Box P3
HER2: Human epidermal growth factor receptor 2
HR: Hazard ratio
NK cells: Natural killer cells
PR: Progesterone receptor
TMA: Tissue microarray
Treg: Regulatory T cells
